# Ultrastructure of the lung in a murine model of malaria-associated acute lung injury/acute respiratory distress syndrome

**DOI:** 10.1186/1475-2875-13-230

**Published:** 2014-06-13

**Authors:** Elizabeth H Aitken, Elnara M Negri, Renato Barboza, Maria RI Lima, José M Álvarez, Claudio RF Marinho, Elia G Caldini, Sabrina Epiphanio

**Affiliations:** 1 Department of Immunology, University of São Paulo, São Paulo, Brazil; 2 Laboratory of Medical Investigation 59, Faculty of Medicine, University of São Paulo, São Paulo, Brazil; 3 Department of Physical and Earth Sciences, Federal University of São Paulo, Diadema, Brasil; 4 Department of Parasitology, University of São Paulo, São Paulo, Brazil; 5 Department of Clinical and Toxicological Analyses, Faculty of Pharmaceutical Sciences, University of São Paulo, São Paulo, Brazil

**Keywords:** Malaria, *Plasmodium*, Acute lung injury (ALI), Acute respiratory distress syndrome (ARDS), Transmission electron microscopy, Murine model

## Abstract

**Background:**

The mechanisms through which infection with *Plasmodium* spp. result in lung disease are largely unknown. Recently a number of mouse models have been developed to research malaria-associated lung injury but no detailed ultrastructure studies of the disease in its terminal stages in a murine model have yet been published. The goal was to perform an ultrastructural analysis of the lungs of mice that died with malaria-associated acute lung injury/acute respiratory distress syndrome to better determine the relevancy of the murine models and investigate the mechanism of disease.

**Methods:**

DBA/2 mice were infected with *Plasmodium berghei* strain ANKA. Mice had their lungs removed immediately after death, processed using standard methods and viewed by transmission electron microscopy (TEM).

**Results:**

Infected red blood cell:endothelium contact, swollen endothelium with distended cytoplasmic extensions and thickening of endothelium basement membrane were observed. Septa were thick and filled with congested capillaries and leukocytes and the alveolar spaces contained blood cells, oedema and cell debris.

**Conclusion:**

Results show that the lung ultrastructure of *P. berghei* ANKA-infected mice has similar features to what has been described in *post-mortem* TEM studies of lungs from individuals infected with *Plasmodium falciparum*. These data support the use of murine models to study malaria-associated acute lung injury.

## Background

Malaria-associated acute lung injury/acute respiratory distress syndrome (malaria associated ALI/ARDS) is a manifestation of severe malaria which can result from infection with a number of *Plasmodium* spp. including *Plasmodium falciparum, Plasmodium vivax* and *Plasmodium knowlesi* (reviewed in [1]). It has a high mortality rate [1], however how and why malaria-associated ALI/ARDS occurs is largely unknown.

Gas exchange in the lungs occurs in the alveoli that are separated by septa in which there is a network of capillaries as well as collagen, muscle cells, leukocytes and other interstitial cells. Gases in the alveoli need to traffic to the blood in the capillaries, through the alveoli epithelial cells and the capillaries’ endothelium. Lung injury generally involves inflammation which can cause damage to the alveolar epithelial and/or capillary endothelial cells causing hypoxemia [2]. Morphological studies can help our understanding of lung disease and malaria and, though light microscopy can provide considerable information, it is limited when it comes to the exploration of fine-structural abnormalities. Ultrastructural analysis by electron microscopy has the potential to provide valuable information about the pathology of malaria-associated ALI/ARDS. Currently there are only a few papers describing lung ultrastructure in malaria [3-5]. These papers, looking at lung injury caused by *P. falciparum* in humans, describe infected red blood cells (iRBCs) in close contact with the endothelium of alveolar capillaries, interstitial oedema and thickening of the septa as well as swelling of the endothelial cells resulting in a narrowing of the capillaries. The capillaries are described as occluded by iRBCs [3,4], non-infected red blood cell (niRBCs), monocytes [3-5], and neutrophils and lymphocytes [4]. Leukocytes are visible in the septa [5] and fibrin in the septa is described as absent, focal or widespread [5]. Taken together these results suggest there might be a role for iRBC: endothelial binding in lung injury, and that the narrowing of the capillaries, oedema and presence of leukocytes are likely the morphological basis for respiratory distress [3,4].

Recently a number of murine models have been published with a focus on the lung and malaria [6-9] and, to date, models have been able to provide evidence that a number of factors, including vascular endothelial growth factor [6], neutrophils [10], platelets [11], haemozoin [12], CD36 dependent iRBC sequestration [9], and sodium transporter activity [7] play a role in disease. Lungs from mice which die of acute lung injury can have very different lung pathologies compared to other murine models such as cerebral malaria seen with C57BL/6 infected with *Plasmodium berghei* strain ANKA, including increased oedema and haemorrhages in the lung [6]. However, although the use of murine models to examine malaria-associated ALI/ARDS is a growing area of research there are no ultrastructural descriptions available for the terminal stage of the disease in a murine model.

In mice, only one short description of electron microscopy observations of the lung in malaria has been published [13]. That study describes the pathology seen early in infection (zero to five days) in *P. berghei-* and *Plasmodium yoelii*-infected mice (the mouse strain was not specified). Observations included aggregation of platelets in the capillaries at day 2, enlarged neutrophils in capillaries, distended cytoplasmic extensions of endothelial cells and alveolar macrophages in the alveolar space at day 4 and swollen endothelial cells with cytoplasmic sheets together with neutrophils occupying the capillary space at day 5 post infection [13].

In this study, the aim was to perform a detailed ultrastructural analysis of the lungs of mice that died with malaria-associated ALI/ARDS. It is hoped that this analysis will aid the comparison of the mouse model with human malaria-associated lung injury in order to better determine the relevancy of murine models when researching lung injury and allow the investigation of the morphological basis of the disease. This study describes the ultrastructure of the lungs of mice that have died with malaria-associated ALI/ARDS using the model of DBA/2 mice infected with *P. berghei* strain ANKA (clone 1.49L) [6].

## Methods

### Animals and infection

Six- to eight-week-old male DBA/2 mice were bred under pathogen-free conditions in isogenic mouse facilities in Department of Parasitology, University of São Paulo, Brazil. The mice were housed five animals per cage in standard polycarbonate cages on wood-shaving bedding. They were fed *ad libitum* and had unrestricted access to water. They were kept under controlled temperature and humidity in a light and dark cycle of 12 hrs each. Mice were intraperitoneally infected with 1×10^6^*P. berghei* ANKA (clone 1.49L) iRBCs, as previously described [6]. All protocols were approved by the Health Committee of the Biomedical Sciences Institute of the University of São Paulo (CEUA n°003 page 98 book2 and CEUA n°054 page 128 book2) established by the Brazilian College of Animal Experimentation (COBEA).

### Transmission electron microscopy

For transmission electron microscopy (TEM) lung tissue was obtained immediately after death from two non-infected (NI) and three ALI/ARDS mice (in ALI/ARDS mice this was at nine days post infection), cut into small pieces (1 mm^3^) and fixed by immersion in 2% glutaraldehyde at 4°C, rinsed in 0.1 M phosphate buffer (pH 7.4), followed by post fixation in 1% osmium tetroxide, and block staining in 1% aqueous uranyl acetate_,_ dehydrated using alcohol. The tissues were embedded in 100 EPON for 48 hrs at 70°C. Ultra-thin sections of 70 nm were cut and stained 2% uranil acetate and lead citrate, and examined under a Jeol JEM 1010 transmission electron microscope.

### Histochemistry analysis

Lung tissue was obtained immediately after death (in infected mice this was between seven to 12 days post infection), fixed in formalin (10%) for 24-48 hrs then placed in ethanol until put in paraffin, sectioned (5 μm) and stained with haematoxylin and eosin (HE), Masson’s Trichrome Stain or Picrosirius Red. Quantitative comparison of thickness of alveolar septa of ALI/ARDS and control lungs was done on slides from five NI and five ALI/ARDS lungs by overlaying a 20 um^2^ grid on a 40-fold objective image of HE-stained tissue and measuring septa width where the grid crossed the tissue in the thinnest direction (50 measurements/image and three images/mouse lung). Masson’s Trichrome-stained slides six NI and five ALI/ARDS lungs were examined under a 20-fold objective for fibrotic changes in the septa. Degrees of fibrosis were from Grade 0, no fibrotic changes and Grade 1 isolated alveolar septa with gentle fibrotic changes to Grade 8 fibrous obliteration as defined by Hubner *et al.*[14]. Observations were confirmed in consecutive Picrosirius Red tissue sections of select mice.

## Results

### Overview

**Figure 1:**
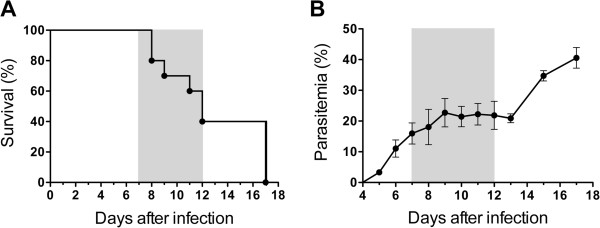
**Infection of DBA/2 mice with *Plasmodium berghei *ANKA.** Survival **(A)** and parasitaemia curves **(B)** from the mice over time, n = 10. The grey area represents the period when the mice die of ALI/ARDS. The experiment presented is representative of five independent experiments.

**Figure 2:**
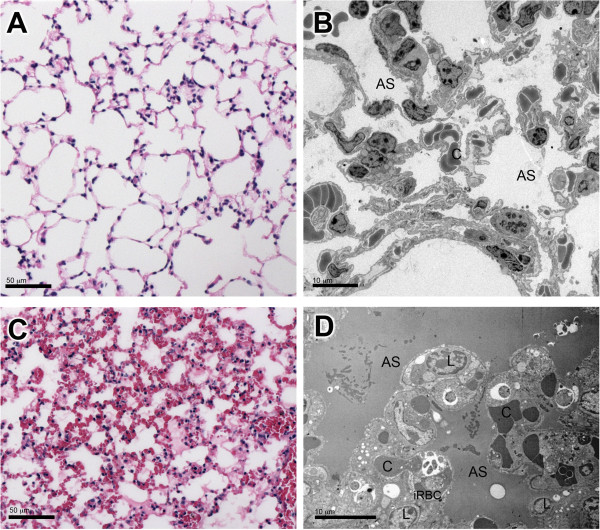
**Lungs from non-infected mice and mice suffering ALI/ARDS.** General morphology of non-infected control lungs **(A and B)** compared to ALI/ARDS lungs **(C and D)**. Seen by light **(A and C)** and transmission electron **(B and D)** microscopy. **(D)** ALI/ARDS lung shows oedema, haemorrhages and thickened septa filled with congested capillaries, infected red blood cell, and leukocytes (iRBC). ALI/ARDS lung, lung from DBA/2 mouse that died with acute lung injury/acute respiratory distress syndrome after infection with *P. berghei* ANKA; AS, alveolar space; C, capillary; L, leukocyte.

DBA/2 mice infected with *P. berghei* ANKA that died of ALI died between seven to 12 days post infection (Figure 1A). An observation of the gross pathology of the lungs revealed pleural effusion and red swollen lungs. Parasitaemia of mice that died of lung injury during this period was typically on average 20% (Figure 1B). HE and TEM images showed that the morphology of ALI/ARDS lungs were completely different when compared with lungs from non-infected mice, with ALI/ARDS lungs showing oedema, haemorrhage and thickened septa filled with congested capillaries, iRBCs and leukocytes (Figure 2).

### Capillaries

**Figure 3:**
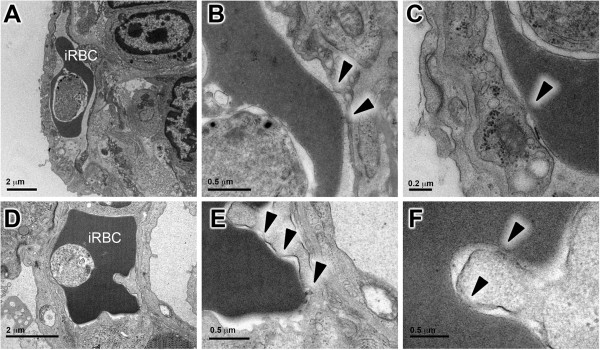
**Infected red blood cell in close contact with endothelium in ALI/ARDS lungs.** Infected erythrocytes were seen in close contact with endothelium in both non-congested **(A, B and C)** and congested capillaries **(D, E and F)**. Bridges between the infected erythrocyte and endothelium could be observed **(B, C, E and F)**. Representative images taken by TEM. ALI/ARDS lung, lung from DBA/2 mouse that died with acute lung injuy/acute respiratory distress syndrome after infection with *P. berghei* ANKA; iRBC, infected red blood cell; arrow heads, points of contact.

**Figure 4:**
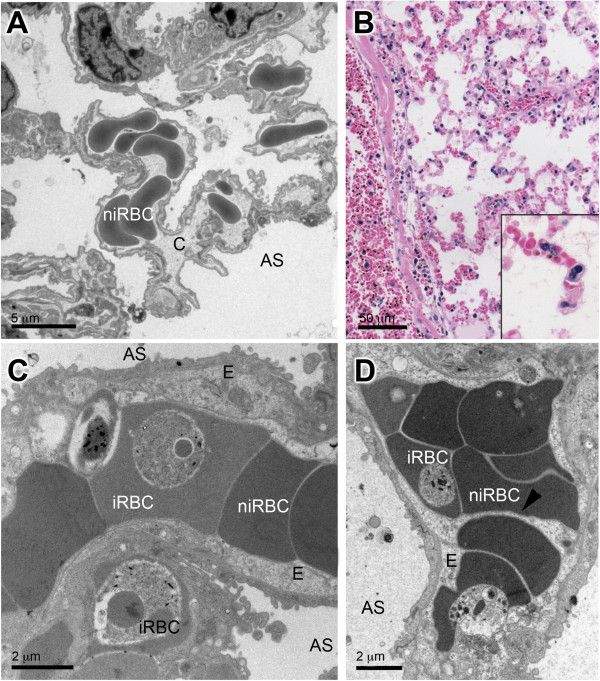
**Congested capillaries and swollen endothelium with extensions.** Image **(A)** shows a non-infected control lung with non-congested capillaries. Congested capillaries in ALI/ARDS lungs could be seen by light **(B)** and TEM **(C and D)** and were filled with iRBC, niRBC and leukocytes (B, insert). Endothelial cells appeared swollen **(C and D)**, and extensions of the swollen cells could be seen as in Figure **(D)** where they are sheltering the red blood cell (arrow head). E, endothelium; niRBC, non-infected red blood cell; AS, alveolar space; iRBC, infected red blood cell. Note: **(A)** is close up of tissue shown in 2B.

**Figure 5:**
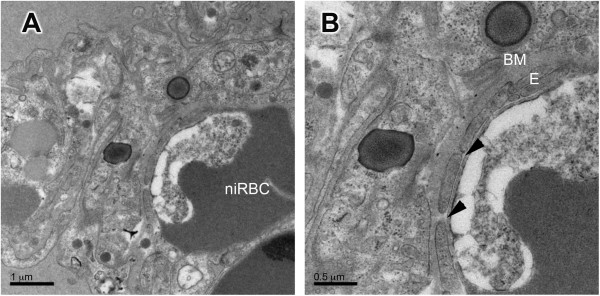
**Fenestrated endothelium and thickening of endothelium basement membrane in ALI/ARDS lung.** Image **(B)** is a close up of **(A)**. TEM images of ALI/ARDS lung, lung from mouse that died with acute lung injuy/acute respiratory distress syndrome after infection with *P. berghei* ANKA. E, endothelium; BM, basal membrane; niRBC, non-infected red blood cell. Fenestrated endothelium are indicated by arrow heads.

TEM of ALI/ARDS lungs showed iRBCs in close contact with the endothelium. This close contact was seen in both non-congested (Figure 3A) and congested capillaries (Figure 3D). In some fields, bridges between the iRBCs and the endothelium were observed (Figure 3B, C, E and F). Congested capillaries seen by light microscopy in HE-stained ALI/ARDS lungs (Figure 4B) were filled with iRBCs, niRBCs and leukocytes (Figure 4B, insert); these observations were confirmed by TEM (Figure 2D, C and D). Some of the congested capillaries showed swollen endothelial cells (Figure 4C and D) and/or thickened endothelial (but not epithelial) basement membrane (Figure 5B). The swollen endothelium included distended cytoplasmic extensions cutting across the alveolar space (Figure 4D). In one field, fenestrated endothelium was observed (Figure 5).

### The septa

**Figure 6:**
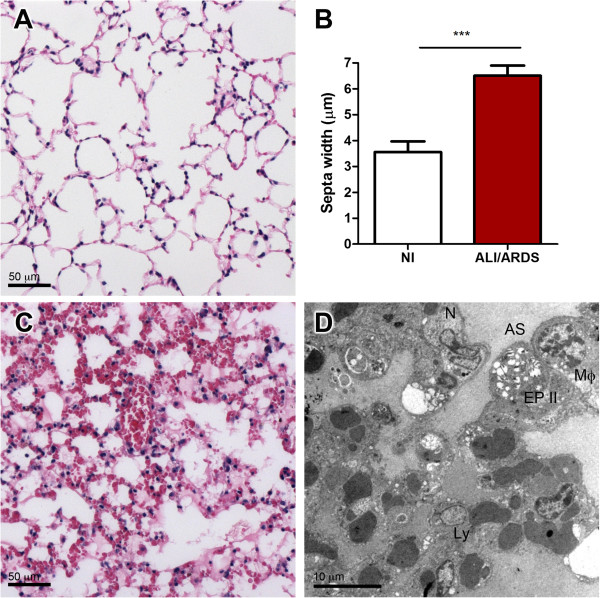
**Thickening of septa in ALI/ARDS lungs.** Light microscopy images of HE-stained, non-infected **(A)** and ALI/ARDS lungs **(C)**. Septa of ALI/ARDS lungs were 80% thicker **(B)** than those of non-infected control lungs, mean (SEM), n = 5/group. ALI/ARDS lung image by TEM, showing oedema and thickened septa containing congested capillaries and leukocytes **(D)**. ALI/ARDS lung, lung from DBA/2 mouse that died with acute lung injury/acute respiratory distress syndrome after infection with *P. berghei* ANKA. Ly, lymphocyte; Mφ, macrophage; N, neutrophil; AS, alveolar space; EPII, epithelium type II.

**Figure 7:**
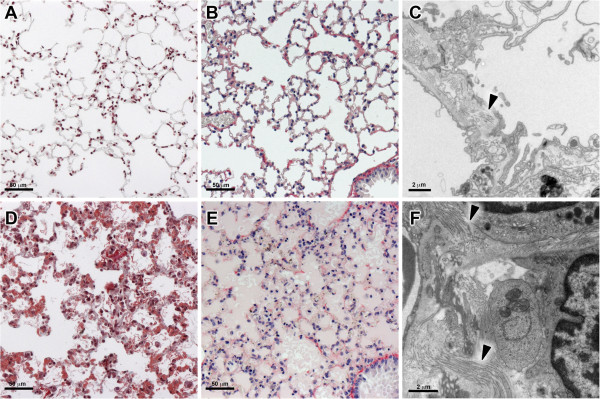
**Absence of fibrosis in ALI/ARDS lungs.** Light microscopy of Masson’s Trichrome stain **(A and D)**, Picrosirius Red **(B and E)** and TEM **(C and F)** of non-infected control **(A, B and C)** and ALI/ARDS **(D, E and F)** lungs. Masson’s Trichrome technique stains collagen blue of the non-infected lung **(A)**. There was no evidence of fibrosis in the ALI/ARDS lungs. Collagen was visible in small, localized areas by TEM in both the non-infected and ALI/ARDS lungs **(B and D)**. ALI/ARDS lung, lung from DBA/2 mouse that died with acute lung injury/acute respiratory distress syndrome after infection with *P. berghei* ANKA. Arrow heads, localized areas of collagen. Note image **(B)** is a close up of tissue shown in 2B.

**Figure 8:**
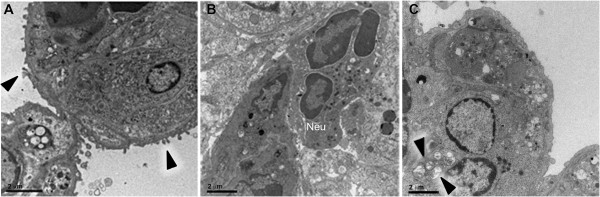
**Septa projections on epithelium, neutrophils and swollen mitochondria.** Septa had projections **(A)** into the alveolar space (arrow heads). Septa were filled with leukocytes **(B)** such as neutrophils (Neu) and cells with swollen mitochondria **(C)** (arrow heads). ALI/ARDS lung, lung from DBA/2 mouse that died with acute lung injuy/acute respiratory distress syndrome after infection with *P. berghei* ANKA.

Quantitative examination of HE-stained lungs showed septa of ALI/ARDS lungs were 80% thicker (6.5 μm ± 0.4 μm) compared to septa of the non-infected controls (3.6 μm ± 0.4 μm) (Figure 6). The thickened septa were not associated with fibrosis as examination of ALI/ARDS lungs and non-infected control lungs stained with Masson’s Trichrome (Figure 7A and D) showed Grade 0 fibrosis (no fibrosis). Additionally, examination of Picrosirius Red-stained ALI/ARDS lungs also showed no fibrosis (Figure 7B and E). Also TEM images of ALI/ARDS and non-infected control lungs showed only small isolated areas with collagen (Figure 7C and F) and there was no obvious proliferation of fibroblasts. Instead TEM images revealed that the septa of ALI/ARDS mice were thickened with a marked leukocyte infiltrate (Figures 2D and 6D), which included macrophages and neutrophils (Figures 6D and 8B). A number of these cells in the septa, such as macrophages, appeared to have phagocytozed erythrocytes (both iRBC and niRBC). Some inflammatory cells in the septa also appeared to have swollen mitochondria (Figure 8C). In many fields the epithelium appeared to have marked projections into the alveolar space (Figure 8A). Neither epithelial apoptosis nor proliferation of epithelial cells was observed.

### Alveolar space

**Figure 9:**
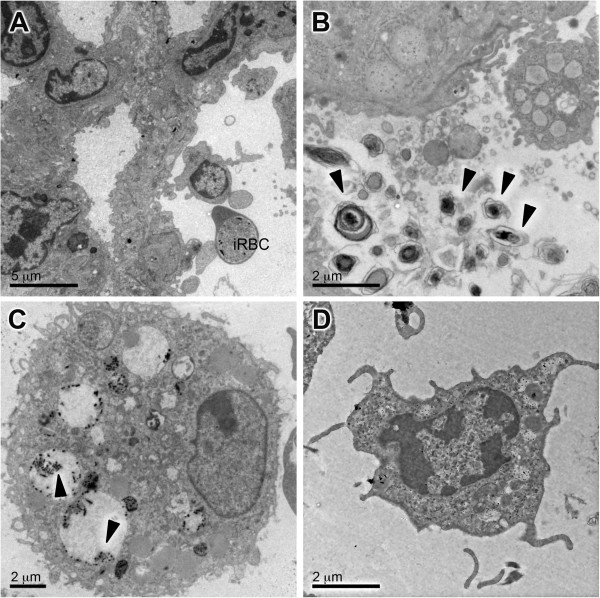
**Cells and cell debris in the alveolar space of ALI/ARDS lung.** TEM images of ALI/ARDS lung showing **(A)** infected red blood cell and leukocyte, **(B)** fragments of surfactant (arrow heads), **(C)** alveolar macrophage with vesicles containing haemozoin (arrow heads) and **(D)** other leukocytes in the alveolar space. ALI/ARDS lung, lung from DBA/2 mouse that died with acute lung injury/acute respiratory distress syndrome after infection with *P. berghei* ANKA.

The alveolar space of ALI/ARDS lungs was filled with oedema (Figure 2D) and also contained cells and cellular debris. These were identified by TEM as iRBCs (Figure 9A), degraded surfactant (Figure 9B) and leukocytes (Figure 9A and D), including alveolar macrophages (Figure 9C). The alveolar macrophage contained crystals, possibly haemozoin (Figure 9C).

## Discussion

This study represents the first description of the lung ultrastructure of mice that have died with malaria-associated ALI/ARDS.

Although ALI/ARDS can be caused by exposure of either the alveoli or the capillaries to a large variety of harmful agents, its progression generally follows certain patterns [15]. Damage in the capillaries is often first seen with sub-endothelial and endothelial oedema, followed by necrosis of endothelial cells, which results in nude basement membrane. More serious injuries often involve the presence of high numbers of inflammatory cells in the septa and damage to the epithelium (primarily epithelium type I), which results in alveolar oedema (often along with hyaline membrane formation). Extensive and prolonged injury or severe epithelial damage is also often associated with stimulation of fibroblasts and collagen deposition (reviewed in [15]).

This study, looking at malaria-associated ALI/ARDS, showed a number of these characteristics. TEM images of infected lungs showed damage to the endothelium, namely endothelial swelling with distended cytoplasmic extensions and thickening of the endothelial basement membrane. The presence of oedema, blood and debris in the alveolar spaces was observed. Although, as in other TEM studies of malaria-associated lung injury, injured epithelium by TEM was not seen [3,4], it must be remembered that this method only looks at a small section of tissue and so it cannot be concluded that epithelial damage is completely absent. Also the presence of oedema seen in the murine model and in *post mortem* cases of malaria-associated lung injury (reviewed in [16]), as well as red blood cells in the alveolar space suggests breakdown of barriers and that epithelial damage is occurring. It is possible that epithelium damage occurs in isolated areas and/or that the oedema is at least partly due to reduced activity of epithelium sodium channels [7] (as opposed to a physical breakdown of the barrier).

Capillary congestion was one of the most prominent observations by both light microscopy and TEM. It is likely that this congestion results in inefficient blood flow and blood gas exchange in the lungs and TEM images provide evidence as to which factors may be contributing to it. When observing iRBCs in close contact with endothelial cells, and in some fields, bridges were seen between the endothelium and iRBCs. This provides evidence that iRBC:endothelium adherence is occurring in the mouse lung, possibly resulting in the accumulation of parasites in the lungs. This observation complements studies which suggest that CD36-dependent iRBC sequestration is related to disease severity by contributing to parasite levels in the lung [17] and murine malaria-associated lung injury [9]. Sequestration may also contribute to pathology by preventing parasite removal by the spleen, promoting parasite growth [18] and activating endothelial cells [19].

In addition to iRBC:endothelium binding, another factor that may contribute to capillary congestion is the endothelium swelling, especially with the endothelium extensions cutting through the capillary lumen. It is hypothesized that the swelling and extensions are an indication of activated endothelium and/or microthrombi organization. *In vitro* studies could provide more information regarding the role of iRBC:endothelium interactions.

The presence of various leukocytes in the lung’s capillaries, septa and alveolar spaces suggests that inflammation plays an important part in the pathogenesis of malaria-associated lung injury in this model. Monocyte/macrophages and neutrophils have been identified previously by electron microscopy in both human [3-5,20] and murine [10,13] malaria-associated lung injury. Monocytes/macrophages are consistently observed in human studies and often contain haemozoin indicating phagocytosis of infected cells [1,3,4,21], and their presence could result in the production of pro-inflammatory products that could also contribute to endothelial injury [22]. The observation of an alveolar macrophage with vesicles filled with crystals (likely haemozoin) suggests that alveolar macrophages play a role in clearing parasites from the alveolar space. Neutrophils have been highlighted as playing a role in severe disease caused by *P. berghei* ANKA, possibly by contributing to pulmonary lesions [10]. In humans however, the role of neutrophils in lung injury during *P. falciparum* malaria still needs to be clarified, as ultrastructural studies of human lungs in severe malaria noted the presence of monocytes [3,4,20] and only one study noted the presence of neutrophils [4]. As lung injury due to other causes is often associated with high numbers of neutrophils in the lungs, the role of neutrophils in *Plasmodium*-induced lung injury in humans should be a research priority in order to clarify whether the mechanisms of disease in non-malaria associated ALI/ARDS can be extrapolated to those of malaria-associated ALI/ARDS.

In humans, lung fibrosis occurs in some but not all individuals suffering from malaria-associated lung injury [23]; no evidence of fibrosis was observed suggesting that this murine model is not suited for investigating the role of fibrosis (if there is any) in malaria-associated lung injury. This may be due to the fact that the mice die before 12 days post infection and instead, mouse models of severe disease that rely on a longer time-frame of infection may be more suitable for looking at fibrosis. Curiously, a fenestrated endothelial cell was observed by TEM, possibly indicating the very beginning of fibrosis in the lungs [24], however it is best not to over-interpret this.

## Conclusion

**Figure 10:**
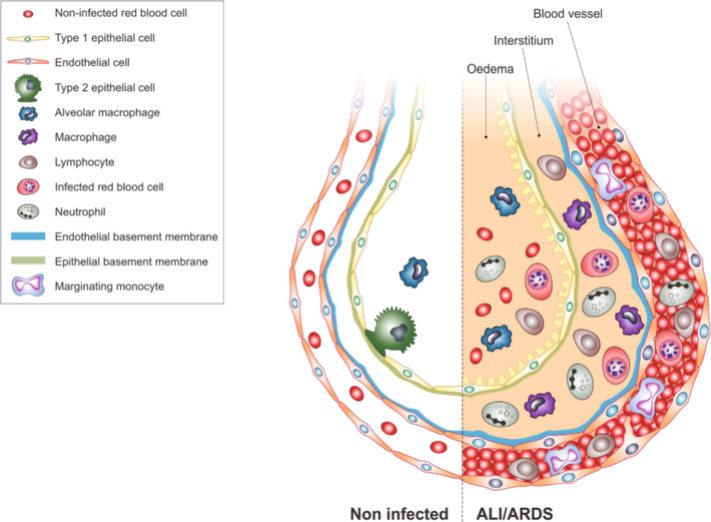
**Diagram showing main findings of the paper.** Using scanning electron microscopy a number of differences are visible in ALI/ARDS mouse lungs when compared to lungs of non-infected mice. In summary the endothelium of the capillaries ALI/ARDS lungs are swollen with distended cytoplasmic extensions and thickened basement membranes. The capillaries themselves are completely congested with leukocytes, niRBC and iRBC, in some instances bridges between iRBC and endothelium surfaces are visible. The alveolar space contains oedema, inflammatory cells and projections from septum epithelium, the septum are thick and full of leukocytes. ALI/ARDS: acute lung injury/acute respiratory distress syndrome. The layout of a single alveoli split to compare non-diseased and diseased lungs is based on a number of previous papers and books [25,16,27].

This is the first detailed ultrastructural analysis of lungs of mice that have died with malaria-associated ALI/ARDS. These results show that iRBC:endothelium binding and endothelium swelling with cytoplasmic extensions are present and likely contribute to the occlusion of the capillaries. Damage to the endothelium was clear with the swelling and thickening of the endothelium basement membrane. In contrast, damage to the epithelium was less obvious and it was not possible to confirm if the alveolar oedema that was observed was due to damage of the epithelial layer. Thickening of septa occurred and was attributed to extensive leukocyte infiltrate, occluded capillaries and cellular oedema, while there seemed not to exist any significant fibrosis. The excessive numbers of leukocytes in the capillaries and septa highlight the role of inflammation in lung injury. A summary of the changes seen by electron microscopy is represented in diagram in Figure 10.

It is shown here for the first time that the lung ultrastructure in mice that died with malaria-associated ALI/ARDS, after infection with *P. berghei* ANKA, has many similar features to what has been described in *post-mortem* TEM studies of lungs from individuals infected with *P. falciparum*. Some of these similarities are the presence of inflammatory cells, oedema, occluded capillaries, swollen endothelium, and iRBC:endothelium contact. Although human malaria-associated lung injury still needs to be further investigated in order to clarify some morphological characteristics of the disease, overall these data support the use of murine models to study malaria-associated lung injury.

## Abbreviations

ALI: Acute lung injury; ARDS: Acute respiratory distress syndrome; HE: Haematoxylin and eosin; iRBC: Infected red blood cell; niRBC: Non-infected red blood cell; TEM: Transmission electron microscopy.

## Competing interests

The authors declare no commercial or other associations that might pose competing interests.

## Authors’ contributions

EHA designed and performed the experiments, discussed the results, analysed the data and wrote the manuscript. RB, SE and EMN designed and performed the experiments, discussed the results and analysed the data. JMA, CRFM, EGC, and SE conceived and designed the study, discussed the results and wrote the manuscript. MRIL reviewed the manuscript. CRFM and SE funded this work. All authors read and approved the final manuscript.
